# Dimensional Analysis Model Predicting the Number of Food Microorganisms

**DOI:** 10.3389/fmicb.2022.820539

**Published:** 2022-02-08

**Authors:** Cuiqin Li, Laping He, Yuedan Hu, Hanyu Liu, Xiao Wang, Li Chen, Xuefeng Zeng

**Affiliations:** ^1^Key Laboratory of Agricultural and Animal Products Storage and Processing of Guizhou Province, Guizhou University, Guiyang, China; ^2^College of Liquor and Food Engineering, Guizhou University, Guiyang, China; ^3^School of Chemistry and Chemical Engineering, Guizhou University, Guiyang, China

**Keywords:** dimensional analysis, Pi theorem, predicting microorganism model, storage time, validation

## Abstract

Predicting the number of microorganisms has excellent application in the food industry. It helps in predicting and managing the storage time and food safety. This study aimed to establish a new, simple, and effective model for predicting the number of microorganisms. The dimensional analysis model (DAM) was established based on dimensionless analysis and the Pi theorem. It was then applied to predict the number of *Pseudomonas* in Niuganba (NGB), a traditional Chinese fermented dry-cured beef, which was prepared and stored at 278 K, 283 K, and 288 K. Finally, the internal and external validation of the DAM was performed using six parameters including *R*^2^, *R*^2^_*adj*_, root mean square error (RMSE), standard error of prediction (%SEP), *A*_*f*_, and *B*_*f*_. High *R*^2^ and *R*^2^_*adj*_ and low RMSE and %SEP values indicated that the DAM had high accuracy in predicting the number of microorganisms and the storage time of NGB samples. Both *A*_*f*_ and *B*_*f*_ values were close to 1. The correlation between the observed and predicted numbers of *Pseudomonas* was high. The study showed that the DAM was a simple, unified and effective model to predict the number of microorganisms and storage time.

## Introduction

At present, predictive microbiological models are desirable tools in the food industry. They combine microbial growth, mathematical model, and statistics (Mcmeekin et al., 2002; Tarlak et al., 2018). These models can assess the dynamic changes in the growth of certain microorganisms in food using microbial predictive methods, providing a sound basis for the rapid assessment and prediction of storage time (shelf life) and food safety. The predictive microbiological models are a valuable tool for managing and guaranteeing food safety and promoting progress in the food industry (McMeekin et al., 2006; Tamplin, 2018).

Predictive microbiological models are classified into primary, secondary, and tertiary models for predicting microbial growth (Whiting and Buchanan, 1993; Isabelle and André, 2006). Primary models do not include factors but focus on the time behavior. Secondary models deal with the response of parameters that appear in primary modeling approaches such as temperature, pH, and so forth (Giannuzzi et al., 1998; Molina and Giannuzzi, 1999; Xiong et al., 1999; Davey, 2010; Zimmermann et al., 2013). The tertiary level combines the first two types of models with user-friendly applications (Dyer, 1996; McKellar and Lu, 2003; Longhi et al., 2014). The growth of microorganisms requires nutrients, but none of the existing models consider the impact of nutrients on the growth of microorganisms. The nutrition of microorganisms in food is the food itself. Therefore, food mass should be included to build a microbial prediction model. Temperature is the key factor affecting microbial growth. In statistical mechanics, the absolute temperature is proportional to the average kinetic energy of the molecules or atoms of a system (Xu et al., 2012; Bormashenko, 2020). Food microorganisms and food are also composed of molecules or atoms. Therefore, the influence of absolute temperature on microorganisms can be regarded as the influence of the average kinetic energy *E*_K_ of the molecules or atoms of the food-microbial system, and this E_K_ is proportional to the absolute temperature θ (Xu et al., 2012; Bormashenko, 2020), that is, *E*_K_ = *cθ*,where *c* is scale coefficient, KJ⋅K^–1^.

From a professional point of view, the main factors affecting the growth of microorganisms are food mass, temperature (reflection of the average translational kinetic energy of internal particles in the system), pH, water activity (*a*_*w*_) (Sautour et al., 2003), and time. Considering the growth of food microorganisms in the environment, the surface area of the food affects the heat and mass transfer between the food and the environment (Mohos, 2017). The large surface area per unit mass of food helps in heat transfer and contributes to microbial growth within the growth temperature range. In the meantime, the surface area affects the mass transfer and the transfer of nutrient molecules or particles. When the temperature of a substance is greater than absolute 0 K, its molecules or atoms have average kinetic energy (Xu et al., 2012; Bormashenko, 2020). Therefore, mass transfer affects the transfer of molecules or atoms, and then influences temperature diffusion. The temperature obviously affects the growth or metabolism of microorganisms. In addition, mass transfer will affect the absorption of substances by microorganisms, thus influencing the metabolism and growth of microorganisms. Therefore, mass transfer affects temperature diffusion and microbial growth or metabolism. Thus, the surface area of food is also used as a factor affecting the growth of microorganisms. Therefore, the main factors influencing microbial growth include food mass, temperature θ, time T, surface area S_T_ at time T, pH_T_ (pH at time T), and a_wT_ (water activity at time T). But how are so many variables put into a model? Considering that the dimensional analysis combined with the π theorem is an effective method to solve multivariate problems, the present study attempted to use dimensional analysis and the π theorem to construct a microbial prediction model.

The dimensional analysis method (Kuneš, 2012; Singh, 2017) is a well-developed methodology in physics, chemical engineering, food engineering, and so on. It is used for diluting complex physical phenomena to the most simplified form (Buckingham, 1914; Chandarana et al., 2010). It reduces the number of variables in the problem by combining dimensional variables to form nondimensional parameters. Moreover, dimensionless groups allow the application of empirical correlation to a wide range of conditions. A classic example of this aspect is discovering the Reynolds number and the relationship between friction coefficient, Reynolds number, and relative roughness in the fields of chemical engineering and food engineering using dimensional analysis. Meanwhile, dimensional analysis is also applied to characterize some indicators in biological systems (Mcmahon, 1973; Pilbeam and Gould, 1974; Ruzicka, 2008). For example, the square of characteristic body dimension *L* must support a weight that increases with *L*^3^; the surface density is *M/L^2^* (kg/m^2^); and elastic criteria impose limits on biological proportions and, consequently, on metabolic rates. However, it is true that biological laws are not derivable from physical laws in any simple sense (Mcmahon, 1973). Therefore, dimensional analysis is seldom used in biology. The dimensional analysis has several advantages, and hence it is necessary to consider its application in predicting microbial growth in food. It can be applied more widely in biology with the development of cognition. Buchanan (2010) commented, “Dimension clearly matters more than we might naively think, and perhaps biology awaits a similar explosion.” However, the predictive model based on the combination of temperature and dimensional analysis on microbial growth has not been reported. Under some conditions, the Pi theorem can also be written in the form of a power function (Nagy, 2019), which is undoubtedly helpful in its application. Therefore, investigating and modeling the microbial growth of foods are important.

Niuganba (NGB) is a traditional Chinese fermented beef (Tian et al., 2021). It has a unique flavor, delicious taste, and nongreasy texture. NGB has a shelf life, and *Pseudomonas* is usually the spoilage bacteria in meat products.

The objective of this study was to introduce dimensionless numbers by dimensional analysis and then to establish a predictive microbiological model based on the Pi theorem. Then, the model was used to predict *Pseudomonas* in NGB during storage. This new unified predictive microbiological model anticipated the microbial growth of foods, as well as the storage time. It might support the development of food microbiology and predict the storage time.

## Materials and Methods

### Materials

Fresh rump, salt, garlic, ginger, fresh red pepper, sesame, tea polyphenols, glucose, peppercorns, monosodium glutamate (MSG), and white liquor were purchased from local supermarkets. *Lactobacillus pentosus* MT-4 (China Center for Type Culture Collection M2016001) was preserved in the laboratory. *Staphylococcus xylosus* American Type Culture Collection 2997 was collected from the Guangdong Industrial Microbiology Collection, China. *Wickerhamomyces anomalus* yeast was purchased from Yichang Angel Yeast Co., Ltd., China. The De Man, Rogosa, and Sharpe (MRS) liquid medium was prepared as described in a previous study (de-Man et al., 1960). The MRS agar medium was prepared by adding agar (20 g/L) to the MRS liquid medium. A yeast extract peptone dextrose (YPED) medium was prepared using peptone (20 g), glucose (10.0 g), yeast extract (10.0 g), and distilled water (1,000 mL). A mannitol salt (MS) medium was prepared using peptone (10.0 g), beef extract (1.0 g), D-mannitol (10.0 g), sodium chloride (75.0 g), phenol red (0.025 g), and distilled water (1,000 mL). The YPED agar medium was prepared by adding agar (20 g/L) to the YPED medium, while the MS agar medium was prepared by adding agar (20 g/L) to the MS medium. A *Pseudomonas* agar medium (Gospavic et al., 2008; ISO, 2010) was prepared using peptone (16.0 g), hydrolyzed casein (10.0 g), anhydrous potassium sulfate (10.0 g), magnesium chloride (1.4 g), glycerol (10.0 mL), *Pseudomonas* medium selection agent (five vials), cephaloridine-fucidin-cetrimide (Oxoid, United Kingdom), agar (20 g), and distilled water (1,000 mL). The pH of the medium was adjusted to 7.0 ± 0.2. All the chemicals used in this study were of analytical grade and commercially available.

### Preparation of Niuganba

#### Preparation of Starters

*Lactobacillus pentosus* MT-4 was anaerobically cultured in the MRS medium at 37°C for 24 h. *W. anomalus* yeast was grown in the YPED medium at 30°C for 24 h, and *S. xylosus* was grown in the MS medium at 37°C for 24 h. After culturing, the number of living cells was measured by spreading them on the corresponding agar medium under the same conditions as the corresponding liquid culture. The cells were harvested at 5,000 × *g* at 4°C for 10 min, washed with sterilized physiological saline three times, and resuspended in sterilized physiological saline. Then, the cell concentration of *L. pentosus* MT-4, *W. anomalus* yeast, and *S. xylosus* was adjusted to 6.0 × 10^7^, 7.0 × 10^7^, and 7.0 × 10^7^ CFU/mL, respectively.

#### Selection and Treatment of Raw Rump

Rump with dark red, long fibers, less fat fascia, elastic luster, natural odor, slightly dry appearance, and nonsticky hand with a “marble pattern” was selected as the raw material based on the National Food Hygiene Standard GB 2707-2005 (Ministry of Health, 2005). The raw rump was sliced along muscle lines, fascia and fat were removed, and the blood was washed out with water. The rump strips were divided into rump chunks of 3 × 2 × 2 cm^3^, which were neat and uniform in thickness and stored at 4°C for no more than 3 h.

#### Inoculation, Fermentation, Baking, and Packaging of Niuganba

The washed and cut rump chunks were soaked in white liquor for about 20 min and inoculated with 0.8% MT-4 (6.0 × 10^7^ CFU/mL), 0.8% *W. anomalus* yeast (7.0 × 10^7^ CFU/mL), and 1% *S. xylosus* (7.0 × 10^7^ CFU/mL). Then, the following ingredients (g/100 g rump) were added to the meat: glucose (1.5), salt (1.5), *Perilla* seed (2), tea polyphenols (0.01), ginger (6), red pepper (0.6), garlic (6) orange peel (1) and pepper (1). Repeated marination was carried out until the surface of the meat was wet and soft. The mixture was then fermented in closed containers at 20°C for 36 h. Subsequently, the fermented NGB was taken out and placed in an oven at 70°C for drying until the moisture content of NGB was approximately 30%. Subsequently, it was cooled to room temperature and packed under vacuum.

### Storage and Sample Analysis

Vacuum-packaged NGB samples were stored in the incubator at 278, 283, and 288 K for different storage times. The samples were randomly taken out at frequent intervals appropriate for each storage time to determine the number of *Pseudomonas* (log_10_ CFU/g), *S*_T_, and pH_T_.

#### Colony Count of Pseudomonas

In the aseptic operation room, each NGB sample (25 g) was homogenized in sterile saline (225 mL). The solution made using sterile saline was spread onto the *Pseudomonas* agar plate and incubated at 30°C for 72 h to determine the number (CFU/g) of *Pseudomonas* (Gospavic et al., 2008; ISO, 2010).

#### Determination of Surface Area and pH

For determining the surface area (*S*), a paper towel was wrapped around NGB samples, and then the area of the paper towel was measured in m^2^ or cm^2^. The pH was determined with a pH meter.

### Principle and Hypothetical Model of Dimensional Analysis

#### Principle of Dimensional Analysis

As mentioned in the introduction, the main factors affecting *N*_T_/*N*_0_ (where *N*_T_ is the number of microorganisms per unit mass of food at a certain time *T*, and *N*_0_ is the initial number of microorganisms per unit mass of food) include food mass *M*, temperature θ (reflection of the translational kinetic energy of the internal molecules or atoms of the food microorganism system), time *T*, surface area *S*_T_ at time *T*, pH_T_ (pH at time *T*), and *a*_wT_ (water activity at time *T*).

When the microorganisms are in lag time (λ). *N*_T_ is obviously equal to *N*_0_. As food is deteriorated by spoilage microorganisms at a later stage, this study focused on the changes in food microorganisms after the lag time. Therefore, *N*_T_/*N*_0_ = *f* (M, *S*_T_, θ, *T-*λ, pH_T_, *a*_wT_) (*T* > λ). *N*_T_/*N*_0_ is obviously dimensionless. The pH_T_, and *a*_wT_ are also dimensionless. M, *S*_T_, θ, and *T-*λ mean food mass (kg), surface area (m^2^), temperature (K), and time (S), respectively. The growth of microorganisms is related to energy. Absolute temperature is proportional to the average kinetic energy of molecules or atoms of a system. As mentioned in the introduction, the influence of food temperature on microorganisms can be regarded as the influence of the average kinetic energy [*cθ*(KJ, *c* is scale coefficient, KJ⋅K^–1^.)] of molecules or atoms in the food–microorganism system. Then, the parameters *M*, *S*_T_, *cθ*, and *T*-λ can be combined into the formula$\frac{c\,\theta \,{\left(T-\lambda \right)}^{2}}{M\,S_{\text{T}}}$, and hence its dimension is expressed as follows:


$$\frac{\text{[KJ}•{\text{K}}^{-1}\times K\times {\text{S}}^{2}]}{\text{[Kg}\times {\text{m}}^{2}]}=\frac{\text{[KJ]}}{\text{[Kg}\times {\text{m}}^{2}\times {\text{S}}^{-2}]}=\frac{\text{[KJ]}}{\text{[KJ]}}={\left[\mathrm{KJ}\right]}^{0}$$

It is a dimensionless number. Let He = $\frac{c\,\theta \,{\left(T-\lambda \right)}^{2}}{M\,S_{\text{T}}}$. Relative to the storage time *T* of microorganisms, λ is usually small. Subsequently, only the case where the storage time *T* is much greater than λ is considered, so *T*-λ is approximately equal to *t*, that is He = $\frac{c\,\theta \,{\left(T-\lambda \right)}^{2}}{M\,S_{\text{T}}}\approx \frac{c\,\theta \,{\text{T}}^{2}}{M\,S_{\text{T}}}$. According to the Pi theorem, *N*_T_/*N*_0_ = φ (He, pH_T_, and *a*_wT_). In natural convection under some conditions, the Nusselt number Nu was correlated by a power function with Reynolds number Re, Prandtl number Pr, and Grashof number Gr; Nu = *C*Re*^m^*Pr*^n^*Gr*^k^* (where *C*, *m*, *n*, and *k* are undetermined constants) (McAdams, 1954; Nagy, 2019). Hence, whether the dimensionless number (*N*_T_/*N*_0_) is correlated by a power function with He, pH_T_, and *a*_wT_ needs exploration. Suppose they are related through the following hypothetical Equation 1:

When the storage time T is much greater than λ,


(1) $$N_{T}/N_{0}=f\left(\mathrm{He},{\mathrm{pH}}_{T},a_{\mathrm{wT}}\right)=j_{1}{\left[\frac{c\,\theta \,{\text{T}}^{2}}{{\mathrm{MS}}_{\text{T}}}\right]}^{n_{1}}{\mathrm{pH}}_{T}^{n_{2}}a_{\mathrm{wT}}^{n_{3}}\left(T\gg \lambda \right)$$

where c and j1 are constants. Under normal circumstances, the food quality M can be considered basically unchanged before the food is spoiled. Hence M is also a constant. Therefore, Equation 1 becomes Equation 2:


(2) $${\text{N}}_{T}=j_{1}\,{\text{N}}_{0}\,{\left[c/M\right]}^{n_{1}}\,{\left[\frac{\theta \,{\text{T}}^{2}}{S_{\text{T}}}\right]}^{{\text{n}}_{1}}\,p\,H_{T}^{{\text{n}}_{2}}\,a_{w\,T}^{{\text{n}}_{3}}$$

Because *N*_0_ is also a constant, let j_1_N_0_[c/M]^*n1*^ = j_0_. Then, Equation 2 becomes:


(3) $${\text{N}}_{T}=j_{0}\,{\left(\theta \,T^{2}/S_{T}\right)}^{{\text{n}}_{1}}\,p\,H_{T}^{{\text{n}}_{2}}\,a_{w\,T}^{{\text{n}}_{3}}$$

where *T* is the time (S), *N*_T_ is the number of microorganisms (CFU/g) at time *T*; θ is the temperature (K); *N*_0_ is the initial number of microorganisms (CFU/g); *S*_T_ is the surface area (m^2^); *j*_0_, *n*_1_, *n*_2_, and *n*_3_, are unknown constants; and *j*_0_ closely correlated with food nutrition and initial microbial concentration.

#### Hypothetical Model for Predicting Microbes in Niuganba

Considering the sealed packaging of NGB, it was believed that *a*_*w*_ changed little, and its effects on the number of *Pseudomonas* remained unchanged within shelf life. Therefore, for simplifying the calculation, the effects of *a*_*w*_ on microbial growth were considered as constant j_0_ in the microbial growth prediction in the subsequent experiment. Thus, Equation 3 became:


(4) $${\text{N}}_{T}=j_{2}\,{\text{j}}_{0}\,{\left(\theta \,T^{2}/S_{T}\right)}^{{\text{n}}_{1}}\,p\,H_{T}^{{\text{n}}_{2}}$$

Let _*j*2*j0*__=__*j*_, then, Equation 4 became:


(5) $${\text{N}}_{T}=j\,{\left(\theta \,T^{2}/S_{T}\right)}^{{\text{n}}_{1}}\,p\,H_{T}^{{\text{n}}_{2}}$$

Let *y* = N*_T_*, *x*_1_ = *θT*^2^/*S*_T_, and *x*_2_ = *pH*_T_. After taking the logarithm of both sides of Equations 5, 6 were obtained:


(6) Logy=Logj+nLogx1+1nLogx22

Both Equations 5, 6 were referred to the dimensional analysis model (DAM).

#### Application of Hypothetical Model Dimensional Analysis Model for Predicting Microbial Growth in Niuganba

The vacuum-packaged NGB was stored at a certain temperature for some days. Then, some samples were randomly taken out (sealed after sampling) to determine the number of *Pseudomonas*, *S*_T_, and pH for predicting microbial growth and storage time.

#### Validation of the Model

In this study, two validation procedures were performed. First, for internal validation, the model was validated against the same data used to build the model (Dong et al., 2007). It ensured that the model accurately described the data from which it was generated and represented any biological trends in the data. For external evaluation (Giffel and Zwietering, 1999), new data from the storage of NGB samples selected randomly within the range of experimental design were used. The accuracy of the models describing microbial growth was evaluated using the following seven criteria: coefficient of determination (*R*^2^), adjusted coefficient of determination (*R*^2^_adj_), median relative error (RE) (Equation 12) of model predictions, root mean square error (RMSE) (Hu et al., 2018; Antunes-Rohling et al., 2019; Park et al., 2020), %SEP, accuracy factor (*A*_*f*_), and bias factor (*B*_*f*_) (Baranyi et al., 1993; Ross, 1996; Tarlak et al., 2018), expressed as Equations 7–13, respectively.


(7) $${\text{R}}^{2}=1-\frac{\sum_{i=1}^{n}{\left(N_{\text{oberseved}}-N_{\text{predicted}}\right)}^{2}}{\sum_{i=1}^{n}{\left(N_{\text{oberseved}}-N_{\bar{\text{oberseved}}}\right)}^{2}}$$

(8) $${\text{R}}_{\text{adj}}^{2}=1-\frac{\left(1-R^{2}\right)\,\left(n-1\right)}{\left(n-N-1\right)}$$

(9) $$\mathrm{RE}=\frac{N_{\text{predicted}}-N_{\text{observed}}}{N_{\text{observed}}}$$

(10) $$\mathrm{RMSE}=\sqrt{\frac{\sum_{i=1}^{n}{\left(N_{\text{observed}}-N_{\text{predicted}}\right)}^{2}}{n}}$$

(11) $$\%\mathrm{SEP}=\frac{100}{\bar{{\text{N}}_{\text{observed}}}}\sqrt{\frac{\sum_{i=1}^{n}{\left(N_{\text{observed}}-N_{\text{predicted}}\right)}^{2}}{n}}$$

(12) $${\text{A}}_{\text{f}}=10^{\left(\sum_{\text{i}=1}^{n}\left|\log\left(N_{\text{predicted}}/N_{\text{observed}}\right)\right|\,/\,n\right)}$$

(13) $${\text{B}}_{\text{f}}=10^{\left(\sum_{\text{i}=1}^{n}\text{l}\,o\,g\,\left(N_{\text{predicted}}/N_{\text{obseerved}}\right)\,/\,n\right)}$$

N_predicted_ and N_observed_ refer to the predicted number of microorganisms (log_10_ CFU/g) and the observed number of microorganisms (log_10_ CFU/g), respectively, and $N_{\bar{\text{observed}}}$ represents the mean of the observed number of microorganisms (log_10_ CFU/g). Also, *n* represents the number of observations, and *N* is the number of variable parameters in the predictive model. The goodness of fit (*R*^2^) and RMSE were used as a quantitative means of measuring the performance of the model. The success of the model in predicting the dependent variables from the independent variables increased when the values of *R*^2^ were closer to 1. The RMSE values approached zero, indicating that the data closely fitted the model. *R*^2^_*adj*_ was based on the squared Pearson correlation coefficient considering the number of experimental points and parameters. Good fits were obtained when *R*^2^_*adj*_ values were almost one.

The *B*_*f*_ estimates a mean variation between the predicted and observed values. *A*_*f*_, which is analogous to RMSE, estimates the mean difference between the predicted and observed values, disregarding whether the difference is positive or negative. A value of 1 for *A*_*f*_ and *B*_*f*_ indicates an exact agreement between predicted and observed values.

### Data Analysis

Each experiment was repeated independently three times, and the data were presented as mean ± standard deviation. Statistical software SPSS 19.0 and Origin 2018 were used for correlation analysis, regression analysis, calculation of the goodness-of-the-fit parameters, and plotting.

## Results

### Application of Dimensional Analysis Model in Niuganba

**FIGURE 1:**
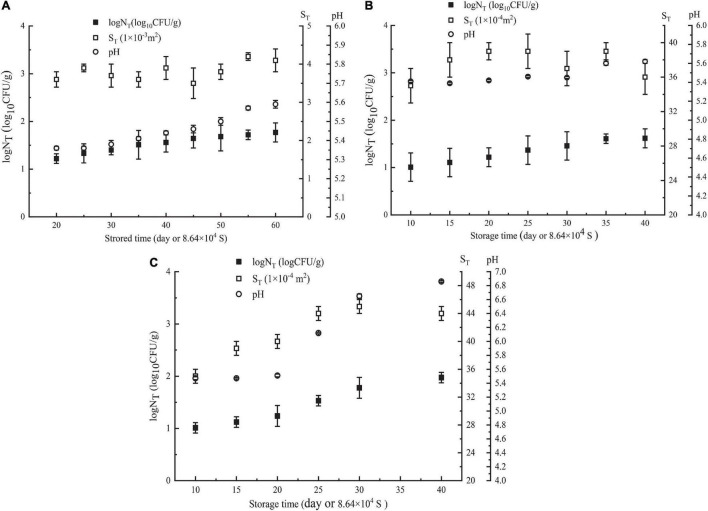
Parameters of NGB (log*N*_T_, *S*_T_, storagetime, and pH values) obtained at different θ. **(A)** θ = 278 K, **(B)** θ = 283 K, and **(C)** θ = 288 K. *N*_T_ means observed *Pseudomonas* number (CFU/g). Error bars represent the standard deviation of three independent samples.

In the first week, *Pseudomonas* in NGB was not detected when it was stored at 5 or 10°C, and the content of *Pseudomonas* detected was very low when it was stored at 15°C (only 2.67 CFU/g). The plate method could not accurately detect such a low content of *Pseudomonas*, that is, for the low content of *Pseudomonas* during the initial first week of storage of NGB, the plate method could not detect it or the detected data were very low, and its accuracy was difficult to meet the requirements. Additionally, as shown in Figure 1, the number of *Pseudomonas* in NGB stored at 5°C for 20 days, 10°C for 10 days, and 15°C for 10 days was 16.6 CFU/g, 10.2 CFU/g, and 10.3 CFU/g, respectively. This indicated that the content of *Pseudomonas* in the first week was very low and difficult to detect accurately. The content of *Pseudomonas* was so low that it would not certainly result in the corruption of NGB in the first week. Therefore, the data of *Pseudomonas* in the first week were unnecessary to include in the model in this study. In addition, during the experiment, the growth characteristics of microorganisms exhibited significant differences in the early and late stages of storage. This was because food spoilage occurred mainly in the late stage of storage, and the early stage was generally short. The microbial growth data were taken just after the early stage.

#### Establishment of Dimensional Analysis Model on the Growth of Pseudomonas in Niuganba

**FIGURE 2:**
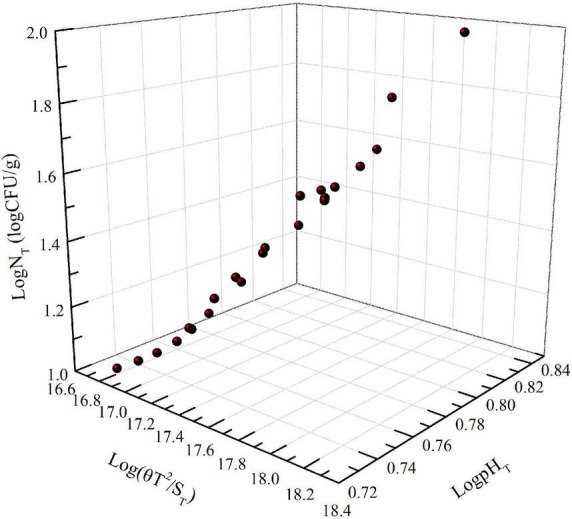
Effect of Log(θT^2^/ S_T_) and Log(pH_T_) on Log(N_T_).


The number of *Pseudomonas* increased with increasing storage time; the effect of storage time on pH showed a similar trend as the number of *Pseudomonas* (Figure 1). This was in agreement with the relationship between the number of microorganisms, pH value, and storage time of this type of food. Moreover, Figure 2 shows that Log*N*_T_ and He number (*θT*^2^/*S*_T_) had a linear relationship with pH_T_. The data in Figure 1 were used in Equation 5. Using SPSS software, Equation 14 for predicting the number of *Pseudomonas* in NGB was obtained.

Logy=-10.445+0.495Logx+14.243Logx2

(14) (R=20.992andR=2adj0.991)

Equation 14 was equivalently changed to Equations 15, 16.


(15) $${\text{N}}_{T}=10^{-10.445}{\left(\theta T^{2}/S_{T}\right)}^{0.495}{\mathrm{pH}}_{T}^{4.243}\left(T\geq 10\mathrm{days},T\gg \lambda \right)$$

(16) $$\mathrm{Therefore},T^{0.99}=\frac{10^{10.445}\,N_{T}}{{\mathrm{pH}}^{4.243}}{\left(\frac{S_{T}}{\theta }\right)}^{0.495}\left(278K\leq \theta \leq 288K\right)$$

where *N*_T_ and *T* are the number of *Pseudomonas* and storage time (second) of NGB, respectively.

The model achieved *R*^2^ values higher than 0.992 and very good *R*^2^_*adj*_ = 0.991. It indicated that the DAM based on the dimensional analysis and the Pi theorem could be used to predict the number of *Pseudomonas N*_T_ and storage time *T* of NGB with high accuracy and precision. Equations 15, 16 were validated as follows.

### Validation of the Dimensional Model

Validation is a vital step for assessing the ability of a new model to interpolate. In this study, *R*^2^, *R*^2^_*adj*_, RE, RMSE, standard error of prediction (%SEP), *A*_*f*_, and *B*_*f*_ were used to evaluate and validate the DAM. *R*^2^ and *R*^2^_*adj*_ were between 0 and a value close to 1, indicating that they were less than 1; and the closer they got to 1, the better, and the smaller the values of | RE|, RMSE, and %SEP, the better.

#### *R*^2^ and *R*^2^_*adj*_ for the Dimensional Model

**FIGURE 3:**
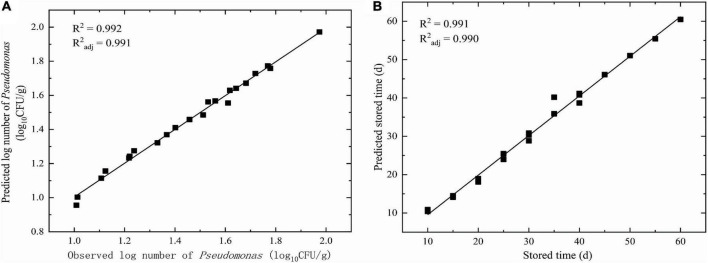
Linear fit for observed and predicted numbers based on DAM for NGB from internal data. **(A)** Observed and predicted numbers of *Pseudomonas*; **(B)** Observed and predicted storage time.

**FIGURE 4:**
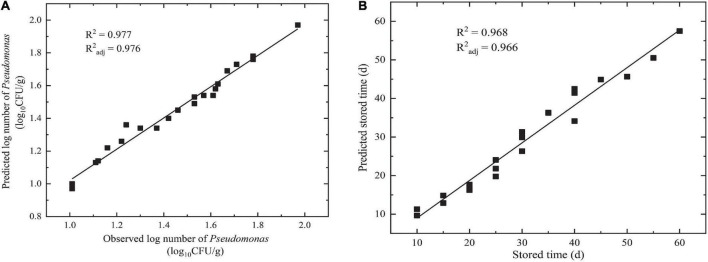
Linear fit for observed and predicted numbers based on DAM for NGB from external data. **(A)** Observed and predicted numbers of *Pseudomonas*; **(B)** observed and predicted storage times.

**FIGURE 5:**
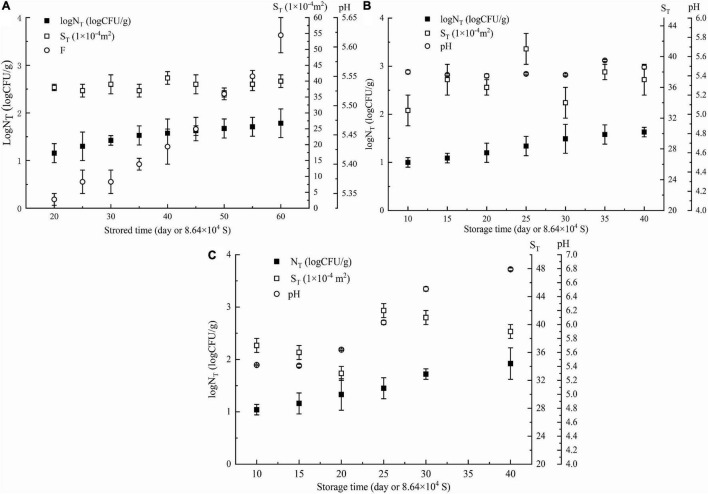
External data: parameters of NGB (log*N*_T_, S_T_, storage time, and pH) obtained at different θ, **(A)** θ = 278 K, **(B)** θ = 283 K, **(C)** θ = 288 K. *N*_T_ means observed *Pseudomonas* number (CFU/g). Error bars represent the standard deviation of three independent samples.

The goodness of fit of the DAM used was evaluated by considering *R*^2^ and *R*^2^_*adj*_ values using Equations 6, 7, respectively. Figures 3, 4 show that the range of *R*^2^ and *R*^2^_*adj*_ of all predicted and observed values was 0.966 − 0.992, irrespective of external (data from Figure 5) or internal verification (data from Figure 1), and observed and predicted values of the microbial growth or storage time. These results indicated that the predicted value was very close or equal to the observed value, suggesting that a DAM could predict the microbial growth (or storage time, shelf life) based on the principles of dimensional analysis and Pi theorem.

#### MRE, Root Mean Square Error, Standard Error of Prediction, A_*f*_, and B_*f*_ for Dimensional Analysis Model

Validation parameters for DAM describing the growth of microorganisms or storage time for NGB, MRE, RMSE, %SEP, *A*_*f*_, and *B*_*f*_, were easily calculated based on the predicted number of *Pseudomonas* and the observed number of *Pseudomonas* in Figures 3, 4, as shown in Table 1. The MRE and RMSE values for validation ranged from 0.60 to 5.31% and from 0.0302 to 2.8846, respectively. The SEP for validation ranged from 2.0838 to 9.4016%. These results indicated that the predictive model yielded the lowest MRE, RMSE, and SEP. Therefore, the predictive model was selected as the best model to fit the number of *Pseudomonas* and the storage time of NGB.

**TABLE 1:** Validation parameters for DAM describing the growth of microorganisms or storage time for NGB.

Sample	Equation	Data	MRE/%	RMSE	%SEP	*A*_f_	*B*_f_
NGB	Equation 15 (predicted log*N*_T_)	Internal	0.60	0.0302	2.0838	1.0127	1.0001
		External	1.60	0.0524	3.6231	1.0228	0.9985
	Equation 16 (predicted storage time)	Internal	2.83	1.4540	4.7388	1.0423	0.9990
		External	5.31	2.8846	9.4016	1.0941	1.0617

Model validation was also performed by considering the bias (*B*_*f*_) and accuracy (*A*_*f*_) using Equation 9, 10, respectively. Ross et al. (2000) reported that the predictive models should ideally have an *A*_*f*_ = 1.00, indicating a perfect model fit, where the predicted and actual response values were equal. The acceptable *A*_*f*_ values were in the range of 1.10–1.15. The *B*_*f*_ value of 1 indicated no structural deviation of the predictive model. Ross (1996) concluded that the range of *B*_*f*_ from 0.9 to 1.05 could be considered perfect for the models, while 0.7–0.9 or 1.06–1.15 was considered to be acceptable, and < 0.7 or > 1.15 was considered to be unacceptable.

The *A*_*f*_ values ranged from 1.0127 to 1.0941, most of which were less than 1.0; the maximum *A*_*f*_ value was less than 1.10. The *B*_*f*_ values for both equations ranged from 0.9985 to 1.0617, all of which were less than 1.06, indicating that the observed data were very close to the equivalence line of fail-safe and fail-dangerous regions. These results revealed that the models could be safely used because the error rates were relatively low.

## Discussion

The prediction of the number of microorganisms is of great value for food quality and safety. The microbial growth model based on dimensional analysis and Pi theorem was presented in this study, considering that dimensional analysis and the Pi theorem are widely applied in physics and chemistry, providing a method for diluting complex phenomena to the most simplified form (Buckingham, 1914; Chandarana et al., 2010): *N*_T_/*N*_0_ = *f*(M, S_T_, θ, T, pH_T_, and *a*_wT_). Also, a hypothetical DAM was established for predicting the number of microorganisms: ${\text{N}}_{T}=j_{1}\,{\text{N}}_{0}\,{\left[c/M\right]}^{n_{1}}\,{\left[\frac{\theta \,{\text{T}}^{2}}{S_{\text{T}}}\right]}^{{\text{n}}_{1}}\,p\,H_{T}^{{\text{n}}_{2}}\,a_{w\,T}^{{\text{n}}_{3}}$*or*${\text{N}}_{T}=j_{0}\,{\left(\theta \,T^{2}/S_{T}\right)}^{{\text{n}}_{1}}\,p\,H_{T}^{{\text{n}}_{2}}\,a_{w\,T}^{{\text{n}}_{3}}$. The DAM reflected the effect of food mass *M* and environmental variables, such as temperature θ, time *T*, initial microbe quantity per unit mass N_0_, surface area *S*_T_, and pH_T_ on *N*_T_. The DAM introduced a dimensionless number He (c*θT*^2^/M*S*_T_), which was an interesting feature of this model. The DAM could be further simplified as${\text{N}}_{T}=10^{-10.445}\,{\left(\theta \,T^{2}/S_{T}\right)}^{0.495}\,p\,H_{T}^{4.243}$, when it was used to predict the number of *Pseudomonas* in sealed NGB. The results showed that DAM had a high *R*^2^_*adj*_. The internal and external verifications confirmed that DAM could be used to predict well the number of *Pseudomonas* and the storage time of NGB. They also validated the hypothesis that the dimensionless number *N*_T_/*N*_0_ was correlated by a power function with the He, pH_T_, and *a*_wT_.

Of course, biologically useful energy also affects microorganism growth. This energy must be stored in food nutrients, and food nutrients are rich relative to a small number of spoilage microorganisms. It can be considered that their content remains basically unchanged before food spoilage. Therefore, their impact on microorganisms can be regarded as a constant and can be combined with the constant term of DAM. In this way, the main contradictions can be grasped and the efficiency of solving problems can be greatly improved. Otherwise, if every aspect is considered, the problem cannot be started and solved.

One advantage of the DAM was that it did not require the initial number of microorganisms. It was emphasized that the value of *N*_0_ was not needed. However, it did not mean that *N*_0_ had no impact on the number of spoilage microorganisms during storage. Its impact on spoilage microorganisms must be the greatest because *N*_T_ was the result of the growth of *N*_0_, that is, ${\text{N}}_{T}\,\mathrm{equaled}\,j_{1}\,{\text{N}}_{0}\,{\left[c/M\right]}^{n_{1}}\,{\left[\frac{\theta \,{\text{T}}^{2}}{S_{\text{T}}}\right]}^{{\text{n}}_{1}}\,p\,H_{T}^{{\text{n}}_{2}}\,a_{w\,T}^{{\text{n}}_{3}}$. Just because *N*_0_ was a constant, it could be incorporated into the constant term of the equation, that is ${\text{N}}_{T}=j_{0}\,{\left(\theta \,T^{2}/S_{T}\right)}^{{\text{n}}_{1}}\,p\,H_{T}^{{\text{n}}_{2}}\,a_{w\,T}^{{\text{n}}_{3}}$. Therefore, this undoubtedly simplified the method of predicting microorganisms. However, microbial prediction models generally required the *N*_0_ value (Park et al., 2020; Tarlak et al., 2020; Yu et al., 2020), which was obtained by direct measurement or curve fitting. Fitting was required at this time because the initial number of spoilage microorganisms in food was usually very low and difficult to detect. However, the fitting usually had errors. Therefore, the model proposed in this study, which did not require the *N*_0_ value (of course, this model did not object to the known *N*_0_), undoubtedly improved the prediction accuracy. Another advantage of this model was that the specific growth rate that was required to be calculated in the general prediction model was not required here (Park et al., 2020; Tarlak et al., 2020; Yu et al., 2020), thereby simplifying the calculation process. Moreover, general models require multiple equations to be combined for prediction. However, one equation of the proposed model could be used to predict the number of microorganisms and the storage time.

Although the number of *Pseudomonas* in NGB was predicted using the DAM, it is conceivable that the DAM can be extended to more general cases: replace NGB with any other food, and replace the *Pseudomonas* with any other microorganism. The growth of any microorganism in any food can be determined using a similar method. The growth inhibition prediction model of any microorganism in any food can also be obtained. Most importantly, the DAM can have a general expression:${\text{N}}_{T}=j_{0}\,{\left(\theta \,T^{2}/S_{T}\right)}^{{\text{n}}_{1}}\,p\,H_{T}^{{\text{n}}_{2}}\,a_{w\,T}^{{\text{n}}_{3}}$. By adjusting the DAM parameters, the prediction model of changes in specific microorganisms in specific food and under specific storage conditions can be established. This unified the prediction model of food microorganisms from the perspectives of biology, physics, and food science. These advantages can undoubtedly help promote the application of the model and reveal the nature of the biophysical mathematical principles behind the growth-inhibitory properties of food microorganisms.

## Conclusion

A DAM based on dimensionless analysis and Pi theorem was introduced in this study. The internal and external verifications suggested the perfect prediction of the number of *Pseudomonas* in sealed NGB and the storage time of NGB, thus proving the rationality and feasibility of this model. An important characteristic of the model was that this model introduced the He number ($\frac{c\,\theta \,{\text{T}}^{2}}{M\,S_{\text{T}}}$). This model could be used to predict both the number of microorganisms and the storage time. Most importantly, based on the same principle, it was also inferred that this model could be used to predict the growth inhibition of any microorganism, and it might represent a universal model based on biology, physics, and food science. These advantages simplified the prediction process. This study laid a strong foundation for applying this model in predicting the number of microorganisms in food products.

## Data Availability Statement

The original contributions presented in the study are included in the article/supplementary material, further inquiries can be directed to the corresponding author/s.

## Author Contributions

CL performed the data analysis and wrote the original manuscript. LH proposed the dimensional analysis model, supervised the experiment, wrote, reviewed and edited the manuscript. YH prepared the NGB. HL performed the experiment of storage. XW performed the data analysis and reviewed the manuscript. LC and XZ reviewed and edited the manuscript. All authors contributed to the article and approved the submitted version.

## Conflict of Interest

The authors declarethat the research was conducted in the absence of any commercial or financial relationships that could be construed as a potential conflict of interest.

## Publisher’s Note

All claims expressed in this article are solely those of the authors and do not necessarily represent those of their affiliated organizations, or those of the publisher, the editors and the reviewers. Any product that may be evaluated in this article, or claim that may be made by its manufacturer, is not guaranteed or endorsed by the publisher.
